# Genome sequences of *Vibrio diabolicus* and *Algoriphagus* sp. isolated from the San Elijo coastal lagoons in San Diego

**DOI:** 10.1128/mra.00478-24

**Published:** 2024-06-25

**Authors:** Kaziah J. Terrell, Rana Petre, Emma K. Stock, Polina Dyadyukova, John A. Kyndt

**Affiliations:** 1 College of Science and Technology, Bellevue University, Bellevue, Nebraska, USA; 2 Erasmus Brussels University of Applied Sciences and Art, Jette, Belgium; 3 Illumina, San Diego, California, USA; University of Southern California, Los Angeles, California, USA

**Keywords:** San Elijo, coastal lagoon, *Vibrio*, *Algoriphagus*, San Diego

## Abstract

We report two draft genome sequences related to the genera *Vibrio* and *Algoriphagus*, isolated from water samples at the San Elijo Lagoon Ecological Reserve, which is one of the largest remaining coastal wetlands in San Diego, California. The initial whole genome-based comparison illustrates the uniqueness of these species.

## ANNOUNCEMENT

Microbial samples were isolated from the San Elijo Lagoon, San Diego, CA (Lat.: 33.004; Long.: 117.271) in September 2023, as part of an ongoing metagenomic sampling project that studies microbiome variations and attempts to identify new microbial species from this distinctive environment. Samples were collected in sterile 50 mL tubes from a depth of 0–10 cm and stored at 4°C. One hundred microliter of the samples was used to inoculate both RCV media agar plates (1), supplemented with biotin (15 µg/L), nicotinic acid (1 mg/L), and 3% NaCl, and nutrient agar (NA) plates supplemented with 2% NaCl. Both were grown aerobically at 30°C. After 1 week of aerobic growth in the light, a white-pinkish colony was picked from NA plates (SE1) and a reddish-pink pigmented colony from RCVBN plates (SE2) and repeatedly transferred to obtain visibly pure cultures. Colonies from these plates were used for genomic DNA extraction using the GeneJet genomic DNA isolation kit (Thermo Scientific). DNA analysis using QuBit and NanoDrop showed a 260/280 absorption ratio of 1.90 (SE1) and 1.79 (SE2).

The sequencing libraries were prepared using the Illumina DNA Library Prep kit and were sequenced by an Illumina MiniSeq using 500 µL of a 1.8pM library. Paired-end (2 × 150 bp) sequencing generated 740,072 reads and 112 Mbps for SE1 and 498,666 reads and 49 Mbps for SE2. Quality control of the reads was performed using FASTQC (version 1.0.0) within BaseSpace (Illumina), using a k-mer size of 5 and contamination filtering. We assembled the genome *de novo* through BV-BRC (2) using Unicycler (v0.4.8) (3). This resulted in a genome of 5,101,831 bp with 41 contigs, 44.8% GC, and an N50 of 694,617 bp for SE1 (25× coverage), and of 4,922,723 bp with 87 contigs, 37.26% GC, and an N50 of 212,341 bp for SE2 (10× coverage). The coarse and fine consistency were 99.8% and 98.3% (*SE1*) and 99.5% and 98.8% (*SE2*) (4). The final assembled genomes were both 100% complete according to CheckM (v1.1.6) (5) with 0% and 0.7% contamination. The genomes were annotated by NCBI PGAP (v6.6) (6), showing that there were 4,594 CDS and 62 tRNAs for *SE1* and 4,073 CDS and 33 tRNAs for *SE2*. Default parameters were used for all software applications unless otherwise noted.

**Fig 1:**
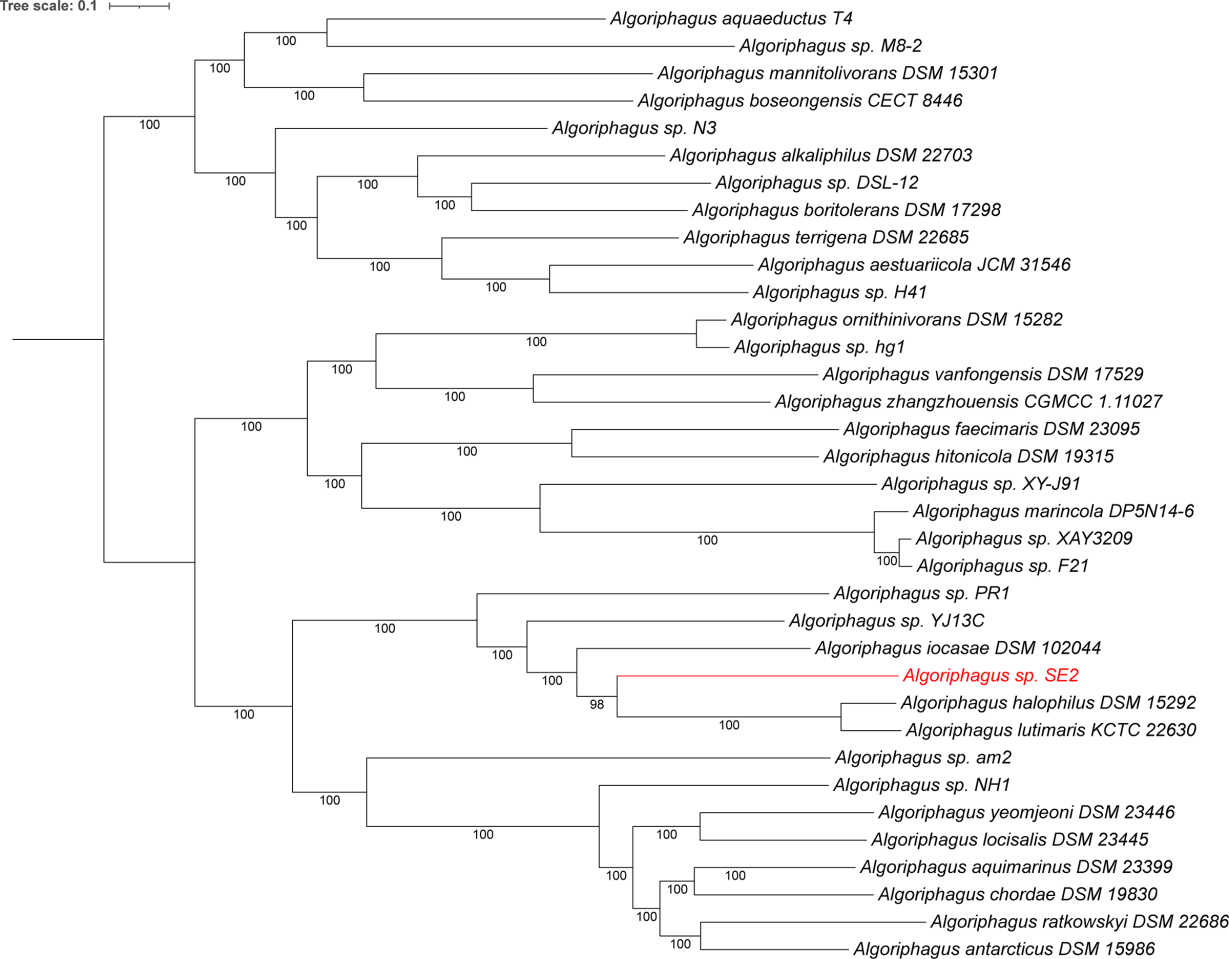
Whole genome-based phylogenetic tree of *Algoriphagus* sp. SE2 compared to representative *Algoriphagus* genomes. The phylogenetic tree was generated using the codon tree method within BV-BRC (2), which used PGFams as homology groups. Nine hundred ninety-nine PGFams were found among these selected genomes using the CodonTree analysis, and the aligned proteins and coding DNA from single-copy genes were used for RAxML analysis (8, 9). One hundred rounds of the “Rapid bootstrapping” option of RaxML were used to generate the support values for the phylogenetic tree. The branch length tree scale is defined as the mean number of substitutions per site, which is an average across both nucleotide and amino acid changes. The new genome is in red. iTOL was used for the tree visualization (13).

The Similar Genome Finder feature using Mash/MinHash (v2.3) in BV-BRC (2, 7) identified SE1 as closest to *Vibrio diabolicus* (545/1000 Kmers), while SE2 gave distant relatives belonging to *Algoriphagus* (10/1,000 Kmers). Whole genome-based phylogenetic analysis was performed using RAxML (v8.2.11) (8, 9) within BV-BRC (2). The best model found by RAxML was Le_Gascuel (10). Consistent with the Mash/MinHash results, these analyses grouped the new genomes closest to *Vibrio* and *Algoriphagus* species (Fig. 1). A JSpecies comparison (v 4.1.1) (11) of average percentage nucleotide identity (ANI) showed that the *Vibrio* genome belongs to *V. diabolicus* with 97.9% ANI to the type strain CNCM I-1629 (12). The *Algoriphagus* genome, on the other hand, is closest to *Algoriphagus iocasae* (77.1%), *Algoriphagus lutimaris* (76.0%), and *Algoriphagus halophilus* (75.8%); however, these are far below the arbitrary species cutoff (95%), which indicates that this is likely a new species and warrants further analysis to determine its taxonomic placement.

## Data Availability

This Whole Genome Shotgun project has been deposited at DDBJ/ENA/GenBank with the following accession numbers JBCLWJ000000000 (*Algoriphagus* sp. SE2) and JBCLWK000000000 (*Vibrio diabolicus* SE1). The versions described in this paper are versions JBCLWJ010000000 and JBCLWK010000000. The raw sequencing reads have been submitted to SRA, and the corresponding accession number are SRR28747447 (*Vibrio diabolicus* SE1) and SRR28772712 (*Algoriphagus* sp. SE2).
